# Long‐term endurance running training and testosterone daily variation in ageing men; a comparative cross‐sectional study

**DOI:** 10.1113/EP093482

**Published:** 2026-09-15

**Authors:** Barbora Bartko, Ľudmila Oreská, Gabriel Buzgó, Genc Berisha, Valentína Zemaníková, Monika Okuliarová, Michal Zeman, Milan Sedliak

**Affiliations:** ^1^ Department of Biological and Medical Sciences Faculty of Physical Education and Sport Comenius University in Bratislava Bratislava Slovakia; ^2^ Department of Health Technologies Faculty of Pharmacy Comenius University in Bratislava Bratislava Slovakia; ^3^ Department of Physical Education Faculty of Natural Sciences Comenius University in Bratislava Bratislava Slovakia; ^4^ Department of Animal Physiology and Ethology Faculty of Natural Sciences Comenius University in Bratislava Bratislava Slovakia

**Keywords:** ageing, circadian rhythm, endurance training, running, testosterone

## Abstract

The aim of this study was to assess the relationship between long‐term endurance running training and daily variation in testosterone, which is known to diminish during ageing, in a comparative, cross‐sectional study. We recruited 45 male participants into four groups [young athletes (YA), young sedentary (YS), master athletes (MA) and elderly sedentary (ES)] and performed anthropometry, strength and ergometry tests. Morning (AM) and afternoon (PM) venous blood samples were collected to evaluate testosterone daily variation, sex hormone binding globulin (SHBG), cortisol and estradiol, and we calculated free testosterone, bioavailable testosterone and the free androgen index. Two‐way and three‐way ANOVAs followed by Bonferroni's multiple comparisons tests were performed. The free testosterone and free androgen index showed a significant PM decrease in the MA group (*P* = 0.0009 and 0.0021, respectively), comparable to YA and YS; however, in the ES group there was no difference between AM and PM values (both *P *> 0.9999). SHBG levels were significantly increased by both the age (*P* = 0.0001) and activity (*P* = 0.0016) factors, leading to a significant decrease in free testosterone, bioavailable testosterone and free androgen index. In the peak power output parameters of ergometry, expressed in both absolute and relative values, MA achieved values comparable to YS, significantly higher than ES (*P* = 0.0020 and *P *< 0.0001, respectively) and lower than YA (*P *< 0.0001). In conclusion, long‐term endurance running training in elderly men might be associated with the presence of daily variation in free testosterone and higher performance levels in comparison to sedentary counterparts, but it might contribute to age‐related changes in SHBG and related testosterone fraction levels.

## Abstract (highlights)

- **What is the central question of this study?**
  Given that testosterone daily variation in men is known to diminish with ageing, we investigated whether endurance running master athletes differ from their sedentary counterparts in the presence of testosterone daily variation.
- **What is the main finding and its importance?**
  Endurance running training might be associated with the presence of testosterone daily variation in ageing men. The presence of this variation in master athletes, comparable to young men, suggests that attenuation of testosterone variation might not be influenced solely by ageing, but also by factors related to inactivity.

## 1 INTRODUCTION

Endurance running is associated with several beneficial physiological adaptations, including improved pulmonary and cardiovascular function, leading to lower resting heart rate and blood pressure (Braschler et al., 2025). On the contrary, there are also common health challenges that endurance‐oriented athletes face. These challenges are now covered by a broader, more multi‐system approach called REDS (relative energy deficiency in sport) syndrome (De Souza et al., 2022; Mountjoy et al., 2018), but in its simpler form they are defined by the male athlete triad model, describing low energy availability, osteoporosis and functional hypothalamic hypogonadism (De Souza et al., 2019; Nattiv et al., 2021).

Although short‐term, moderate‐intensity endurance training in untrained men can increase plasma testosterone concentrations (Grandys et al., 2009), high volumes of exercise in endurance‐trained men have been reported to alter the hypothalamic–pituitary–testicular axis (Hackney, 2001), leading to lower basal free and total testosterone concentrations (Hackney, 2008). Although low serum testosterone concentrations alone do not necessarily imply a maladaptation, they can be accompanied by symptoms of hypogonadism, including fatigue, sexual dysfunction or low bone mineral density, and potentially contribute to adverse health outcomes in athletes (Hooper et al., 2018).

The process of ageing also causes a decline in testosterone levels in men. This relationship is associated with increased risk of cardiovascular diseases, metabolic syndrome, depression, physical frailty or changes in cognitive functioning (Panizzon et al., 2013). Regular physical activity can play a key role in the prevention of chronic diseases typically associated with ageing and improve frailty‐related symptoms and overall quality of life (Izquierdo et al., 2021). Regular exercise is associated with healthy ageing by maintaining physiological integrity and delaying age‐related deterioration in physical function and body composition (Lazarus & Harridge, 2018). However, given that chronic endurance running training in master athletes (MA) is likely to exceed the recommended volumes of exercise during ageing (Izquierdo et al., 2025), these athletes might be influenced, besides the adverse effects of the ageing processes, also by the potential risks of long‐term endurance training.

Ageing also affects the human circadian system, whether as a direct cause of rhythm changes or whether these changes are caused by other pathological processes associated with ageing (Hood & Amir, 2017). These changes mainly consist of rhythm amplitude reduction and phase advance (Duffy et al., 2015; Hood & Amir, 2017; Panagiotou et al., 2021). Regular exercise can function as a Zeitgeber for circadian rhythms, with the ability to reset disruptions in circadian pacemakers (Gabriel & Zierath, 2019; Haupt et al., 2021; Martin et al., 2023); however, much less is known about how physical training influences circadian rhythms of physiological and metabolic processes during ageing (Pradhan et al., 2024). Testosterone is well known for its circadian rhythmicity, with high morning concentrations followed by a decline throughout the day (Shlykova et al., 2020). This rhythmicity tends to attenuate with increasing age (Brambilla et al., 2009), along with a decrease in overall testosterone production (Basaria & Dobs, 1999). It was reported that the variance in average testosterone levels might be under significant genetic influence, and the overall daily intra‐individual variability of free testosterone in men, although partly influenced by genetics, appears to be primarily attributable to individual specific environmental factors (Panizzon et al., 2013).

Our goal was to explore the relationship between long‐term endurance running training and testosterone daily variation in male MA, based on the assumption that regular, chronic exercise, functioning as a Zeitgeber, might contribute to the persistence of testosterone daily variation during ageing, in comparison to elderly men with a sedentary lifestyle. We also assessed the testosterone daily variation in young control groups: both endurance running athletes and their sedentary counterparts. Additionally, we examined the impact of both age and endurance running training on body composition, muscle strength, cardiorespiratory fitness, production of sex‐hormone binding globulin (SHBG) and selected hormones: besides testosterone in its total, free and bioavailable form, we also evaluated estradiol and cortisol, as other well‐known hormones with circadian production.

## 2 MATERIALS AND METHODS

This study was approved by the Ethical Committee of the University Hospital Bratislava–Hospital of Ladislav Dérer, the Academician (number 31/2020) following the ethical standards of the *Declaration of Helsinki* (2000) and its later amendments (World Medical Association, 2001). The study is registered under the ClinicalTrials.gov, registration number NCT05053282. All participants were informed, verbally and in writing, about the study design and procedures, including the possible risks, and subsequently signed an informed consent.

### 2.1 Participants

Healthy male participants from the Bratislava region, Slovakia were recruited via flyers, social media and webpages. Exclusion criteria for this study included recent or current infection, physical or mental disabilities, malignant, cardiovascular, metabolic or autoimmune diseases, malnutrition and pharmacological interference (e.g., steroids, non‐steroidal anti‐inflammatory agents, immunosuppressive and antineoplastic drugs). The participants had to have a body mass index (BMI) within the range of 18.5–35 kg/m^2^.

A total of 45 recruited volunteers were divided into four groups according to their age and training activity. The first group consisted of young endurance‐trained athletes (young athletes, YA; *n* = 13); the second group consisted of young participants with a sedentary lifestyle (young sedentary, YS; *n* = 12); the third group consisted of master endurance‐trained athletes (master athletes, MA; *n* = 11); and the fourth group consisted of elderly participants with a sedentary lifestyle (elderly sedentary, ES; *n* = 9). All participants fell into two age categories: 20–35 years for young and 65–78 years for elderly participants.

To be involved in this study, the participants recruited for the athletic groups (YA and MA) had to regularly perform a running activity for >300 min/week for ≥3 years (YA) or ≥15 years (MA). During the recruitment process, we selected the best runners according to their 10 km and/or marathon race time for each group.

The participants recruited for the sedentary groups (YS and ES) had no history of regular physical training and practice characterized as >150 min of moderate activity or >75 min of vigorous activity per week, which is the minimum physical activity for maintaining health recommended by the World Health Organization (Bull et al., 2020).

### 2.2 Sample and data collection

The design and a complete protocol of our comparative cross‐sectional study has been described in published protocols (Berisha et al., 2023; Vajda et al., 2022). Sample size calculation was performed a priori using G*Power v.3.1.9.2 software based on maximal oxygen uptake (${\dot{V}}_{O_{2}\max}$), selected as a key outcome parameter. The calculation was based on the expected difference in ${\dot{V}}_{O_{2}\max}$ between groups derived from pilot data. Assuming a significance level of α = 0.05 and a statistical power of 0.9, the required sample size was estimated as 14 participants per group (Vajda et al., 2022).

The participants underwent a 3 week testing protocol. During the first week, participants completed two testing sessions on separate days, arranged according to the participants’ availability: one for familiarization and anthropometric measures and one for the physical performance testing (muscle strength and ergometry). The second week served as a monitoring period for the physical activity and dietary tracking (data not shown) in domestic conditions. During the third week, usually on Thursdays, the participants underwent two venous blood samplings, 13 days after the physical performance testing session. All the participants were asked to avoid any intense physical activity before the sample collection. This meant no strenuous physical activity for 48 h prior to sampling for the sedentary groups, and only standardized light‐intensity running training for 24 h prior to sampling for the athletic groups. Apart from these two days before the sampling session, the physical or training activity of the participants was not adjusted during the testing period.

The primary outcome of this study was the daily variation of testosterone in endurance‐running MA >65 years old, compared with their sedentary counterparts and young control subjects. Furthermore, the total testosterone levels and calculated levels of free testosterone, biologically available testosterone and free androgen index are reported, along with evaluation of the SHBG level results.

As secondary outcomes, evaluation of the daily variation in cortisol levels and the values of measured estradiol are reported. Anthropometrics, muscle strength and ergometry measures are included as secondary outcomes for the purpose of group characterization.

#### 2.2.1 Anthropometry

Body height was measured and determined to the nearest centimetre on a digital free‐standing stadiometer (InBody BSM 170, InBody USA). Body weight, along with parameters of relative (percentage) and absolute (kg) body fat, were measured on a bioelectrical impedance‐based digital scale (Body 230, InBody USA), while barefoot and wearing underwear. We evaluated the parameters of body height, body mass, absolute body fat (kg), relative body fat (as a percentage) and BMI.

#### 2.2.2 Muscle strength

To determine the muscle strength of the participants, we measured maximal voluntary contraction during isometric knee extension and flexion on a knee dynamometer (ARS dynamometry, S2P Ltd, Ljubljana, Slovenia). We evaluated the parameters of maximal voluntary torque produced by both legs (bilaterally) during knee extension (Ext Max) and flexion (Flex Max) and their relative values to body mass [relative torque E (RTE) and Relative torque F (RTF), respectively].

Before both knee isometric extension and flexion tests, the participants performed two warm‐up trials of 3–5 s duration and 30 s rest interval with a perceived intensity between 50% and 80% of the maximal effort.

For the tests, three trials were performed with maximal voluntary effort. The participants were instructed to push or pull as fast and hard as possible for 5 s against the lever arms, while being verbally encouraged for the best effort. The rest interval between trials was 90 s, and the rest interval between the extension and flexion measures was 3 min. The best trial with the highest achieved values was then saved for further analysis.

#### 2.2.3 Ergometry

The cardiorespiratory fitness of the participants was determined by a graded cycle ergometry test in the room with standard temperature and humidity. The test was guided by a sports medicine physician.

The testing protocol started with 3 min warm‐up at a workload of 40 W, followed by a gradual increase in workload by 20 W/min every 1 min, performed with maximal effort until voluntary exhaustion or clinical termination.

The heart rate was monitored continuously for the testing duration by an electrocardiographic recording system (Quark T12, Cosmed, Italy). The blood pressure was measured before and after the testing (Metronik BL‐6, STOLL Medizintechnik, Germany). From the cycle ergometry testing, we evaluated the parameters of maximal heart rate (HR Max) peak power output (P Max), P Max relative to body mass and predicted maximal oxygen consumption normalized to body mass ($p{\dot{V}}_{O_{2}\max}$). The value of $p{\dot{V}}_{O_{2}\max}$ was estimated indirectly from the peak power output (P Max) achieved during a continuous incremental cycling test using the American College of Sports Medicine metabolic equation for leg cycling (American College of Sports Medicine, 2025). Cycle ergometry testing in our study protocol was chosen for its safety, considering the elderly population. The advantage of the selected method using cycle ergometry is also its non‐specificity for both endurance runners and sedentary participants, and it enables more objective comparison of the groups. However, because $p{\dot{V}}_{O_{2}\max}$ was estimated indirectly and assessed using a cycle ergometry protocol in a population of endurance runners, this secondary outcome should be interpreted with caution. Although statistically analysed, it is presented primarily to support group characterization and is not used as a basis for the main conclusions of the study.

#### 2.2.4 Blood sample collection and processing

Venous blood samples were collected in the morning (between 07:30 and 08:00 h, in the fasted state) and the afternoon (≥8 h after the morning sampling, between 15:30 and 16:00 h). The procedures were performed by a nurse and a medical doctor. Between the procedures, the participants were instructed to eat standard meals at 10:00 and 13:00 h, with water consumption allowed at any time before or during sampling.

Blood samples were collected into K_3_EDTA and serum tubes. Blood samples were centrifuged (2500*g* at 12°C for 15 min) to separate plasma/serum, which was stored in aliquots at −80°C until further analyses.

### 2.3 Hormone and biochemistry analysis

Plasma concentrations of cortisol, testosterone and SHBG were measured using ELISA kits (DRG Instruments, Germany), according to the manufacturer's instructions. The kits had the following specifications: Cortisol ELISA, EIA‐1887; testosterone ELISA, EIA‐1559; and SHBG ELISA, EIA‐2996. All samples were measured in two assays for each hormone. The intra‐assay variation coefficients were <8%, and the inter‐assay variation coefficients were <14%. The detection limits were 1.3 ng/mL for cortisol, 0.29 nmol/L for testosterone and 0.41 nmol/L for SHBG. Serum estradiol levels were analysed only in the morning samples by a commercial medical laboratory (Unilabs Slovakia, s.r.o.). Serum albumin levels were measured using a commercial colorimetric kit adapted for a 96‐well plate (ALB 500, Erba Lachema, Brno, Czech Republic), following the manufacturer's instructions.

### 2.4 Data analysis

The calculations, statistical analysis and graphical illustrations of the data were done using GraphPad Prism v.8.0.1 (GraphPad Software, USA). All the data were tested for normal or lognormal distribution with Shapiro–Wilk and D'Agostino–Pearson tests, followed by data transformation, if necessary. Outliers identified by the software using the ROUT method (robust regression and outlier removal) were removed from the analysis only after individual evaluation by two independent researchers.

The data were analysed by two‐way ANOVA for the factors of age (young and elderly) and activity (athletes and sedentary), followed by Bonferroni's multiple comparisons tests. The venous blood parameters, in which we also observed differences between the morning (AM) and afternoon (PM) collection, were analysed by mixed design three‐way ANOVA (for the factors of age, activity and repeated measure of sampling time). For clearer data presentation and more detailed interpretation, with the focus on the daily variation of hormones within the age groups, we also analysed these data using two‐way repeated‐measures (RM) ANOVA (for the factors of time and activity) for each age group separately, as biologically targeted follow‐up analyses. We used Bonferroni's multiple comparisons test for each analysis. The results are presented as the mean ± SD. The level of statistical significance was set at *P *< 0.05. The *F*‐value, degrees of freedom, *P*‐value and calculated effect size (partial η^2^) are reported for each ANOVA main effect. The effect size was calculated using the formula: Sum‐of‐squares effect/(sum‐of‐squares effect + sum‐of‐squares error). Predicted mean difference (Mean diff), 95.00% confidence interval (95% CI) and adjusted *P*‐value (*P*) are reported for each *post hoc* comparison test.

## 3 RESULTS

### 3.1 Hormone levels

For the parameters derived from the blood sample analysis, five participants had to be removed from further analyses because of missing data owing to unsuccessful sampling of one or both blood samples. The numbers of participants in the groups were therefore YA *n* = 11, YS *n* = 10, MA *n* = 9 and ES *n* = 9.

All the main effect and interaction results of three‐way ANOVAs are listed in Table 1. The results of *post hoc* multiple comparisons of the three‐way ANOVAs with a significant interaction between factors are listed in Table 2.

**TABLE 1:** Results of the mixed design three‐way ANOVA: The main effects and interactions for the factors of age, activity and repeated measure of sampling time.

Factor	Testosterone (nmol/L)	Cortisol (ng/mL)	SHBG (nmol/L)
Time	***F*(1.35) = 66.71,** *P *< 0.0001, partial η^2^ = 0.656*	***F*(1.35) = 93.61,** *P *< 0.0001, partial η^2^ = 0.728*	*F*(1.34) = 0.1692, *P* = 0.6834, partial η^2^ = 0.005
Age	*F*(1.35) = 1.192, *P* = 0.2824, partial η^2^ = 0.101	*F*(1.35) = 0.8343, *P* = 0.3673, partial η^2^ = 0.050	***F*(1.34) = 18.54,** *P* = 0.0001, partial η^2^ = 0.909*
Activity	*F*(1.35) = 0.9997, *P* = 0.3242, partial η^2^ = 0.086	*F*(1.35) = 1.137, *P* = 0.2937, partial η^2^ = 0.067	***F*(1.34) = 11.74,** *P* = 0.0016, partial η^2^ = 0.864*
Time × Age	*F*(1.35) = 2.978, *P* = 0.0932, partial η^2^ = 0.078	*F*(1.35) = 1.599, *P* = 0.2145, partial η^2^ = 0.044	*F*(1.34) = 1.334, *P* = 0.2561, partial η^2^ = 0.038
Time × Activity	*F*(1.35) = 3.816, *P* = 0.0588, partial η^2^ = 0.098	*F*(1.35) = 0.6593, *P* = 0.4223, partial η^2^ = 0.018	*F*(1.34) = 0.0091, *P* = 0.9245, partial η^2^ = 0.0003
Age × Activity	*F*(1.35) = 0.081, *P* = 0.7782, partial η^2^ = 0,008	*F*(1.35) = 0.2895, *P* = 0.5940, partial η^2^ = 0.018	*F*(1.34) = 0.02328, *P* = 0.6325, partial η^2^ = 0.112
Time × Age × Activity	*F*(1.35) = 2.395, *P* = 0.1307, partial η^2^ = 0.064	*F*(1.35) = 0.3797, *P* = 0.5417, partial η^2^ = 0.011	*F*(1.34) = 2.385, *P* = 0.1317, partial η^2^ = 0.066
	**Free androgen index (%)**	**Free testosterone (nmol/L)**	**Biologically available testosterone (nmol/L)**
Time	***F*(1.34) = 45.33,** *P *< 0.0001, partial η^2^ = 0.576*	***F*(1.34) = 53.43,** *P *< 0.0001, partial η^2^ = 0.611*	***F*(1.34) = 56.24,** *P *< 0.0001, partial η^2^ = 0.6232*
Age	***F*(1.34) = 27.99,** *P *< 0.0001, partial η^2^ = 0.785*	***F*(1.34) = 14.81,** *P* = 0.0005, partial η^2^ = 0.546*	***F*(1.34) = 22.38,** *P *< 0.0001, partial η^2^ = 0.6700*
Activity	***F*(1.34) = 17.68,** *P* = 0.0002, partial η^2^ = 0.697*	***F*(1.34) = 8.213,** *P* = 0.0071, partial η^2^ = 0.4002*	***F*(1.34) = 12.76,** *P* = 0.0011, partial η^2^ = 0.5365*
Time × Age	*F*(1.34) = 3.759, *P* = 0.0609, partial η^2^ = 0.0995	*F*(1.34) = 3.731, *P* = 0.0618, partial η^2^ = 0.099	*F*(1.34) = 3.420, *P* = 0.0731, partial η^2^ = 0.0914
Time × Activity	*F*(1.34) = 2.570, *P* = 0.1182, partial η^2^ = 0.070	*F*(1.34) = 2.722, *P* = 0.1082, partial η^2^ = 0.074	*F*(1.34) = 3.553, *P* = 0.0680, partial η^2^ = 0.0946
Age × Activity	*F*(1.34) = 0.4212, *P* = 0.5207, partial η^2^ = 0.069	*F*(1.34) = 0.0401, *P* = 0.8424, partial η^2^ = 0.003	*F*(1.34) = 0.07007, *P* = 0.7928, partial η^2^ = 0.0063
Time × Age × Activity	***F*(1.34) = 5.095,** *P* = 0.0305, partial η^2^ = 0.130*	***F*(1.34) = 4.931,** *P* = 0.0331, partial η^2^ = 0.127*	*F*(1.34) = 0.4024, *P* = 0.0529, partial η^2^ = 0.1059

*Note*: *n* values represent the number of participants in each of the groups (YA‐young athletes, YS‐young sedentary, MA‐master athletes and ES‐elderly sedentary). For the parameters of testosterone and cortisol, YA *n* = 11, YS *n* = 10, MA *n* = 9 and ES *n* = 9; for the parameters of sex hormone binding globulin (SHBG), free androgen index, free testosterone and biologically available testosterone, YA *n* = 10, YS *n* = 10, MA *n* = 9 and ES *n* = 9.

* *p* < 0.05.

**TABLE 2:** Results of Bonferroni's multiple comparisons test following the mixed‐design three‐way ANOVAs for the factors of sampling time, age and activity.

Comparison	Free androgen index			Free testosterone		
	Predicted mean difference	95% Confidence interval	Adjusted *P*‐value	Predicted mean difference	95% Confidence interval	Adjusted *P*‐value
AM:YA vs. PM:YA	0.1376*	0.03495 to 0.2402*	0.0028*	0.1608*	0.04989 to 0.2717*	0.0010*
AM:YS vs. PM:YS	0.1600*	0.05740 to 0.2626*	0.0004*	0.1820*	0.07107 to 0.2929*	0.0002*
AM:MA vs. PM:MA	0.1485*	0.04033 to 0.2566*	0.0021*	0.1715*	0.05461 to 0.2884*	0.0009*
AM:ES vs. PM:ES	0.01602	−0.09213 to 0.1242	>0.9999	0.02796	−0.08894 to 0.1449	>0.9999
AM:YA vs. AM:YS	−0.1864*	−0.3496 to −0.02327*	0.0141*	−0.1051	−0.2519 to 0.04173	0.4493
AM:MA vs. AM:ES	−0.06210	−0.2341 to 0.1099	>0.9999	−0.01036	−0.1651 to 0.1444	>0.9999
PM:YA vs. PM:YS	−0.1640*	−0.3271 to −0.0008240*	0.0479*	−0.08390	−0.2307 to 0.06291	>0.9999
PM:MA vs. PM:ES	−0.1946*	−0.3665 to −0.02260*	0.0156*	−0.1539	−0.3087 to 0.0008336	0.0523
AM:YA vs. AM:MA	0.1621	−0.005512 to 0.3297	0.0660	0.1070	−0.04378 to 0.2579	0.4683
AM:YS vs. AM:ES	0.2864*	0.1188 to 0.4540*	<0.0001*	0.2018*	0.05093 to 0.3526*	0.0021*
PM:YA vs. PM:MA	0.1730*	0.005418 to 0.3406*	0.0379*	0.1178	−0.03306 to 0.2686	0.2834
PM:YS vs. PM:ES	0.1424	−0.02518 to 0.3100	0.1691	0.04775	−0.1031 to 0.1986	>0.9999

*Note*: YA (young athletes) *n* = 10, YS (young sedentary) *n* = 10, MA (master athletes) *n* = 9 and ES (elderly sedentary) *n* = 9. The *n* values represent the number of participants in each of the groups. AM‐ morning samples, PM‐ afternoon samples

* *p* < 0.05.

Analysis of total testosterone levels by three‐way ANOVA revealed a significant main effect of sampling time factor (Table 1), with lower PM levels compared with AM. The two‐way RM ANOVA of the young groups (YA and YS; Figure 1a) also revealed a significant main effect of sampling time [*F*(1.19) = 43.01, *P* < 0.0001, partial η^2^ = 0.6937], with lower PM levels compared with AM and with no effect of the activity factor [*F*(1.19) = 0.7583, *P* = 0.3947, partial η^2^ = 0.1120] or the interaction [*F*(1.19) = 0.07237, *P* = 0.7908, partial η^2^ = 0.0038]. The analysis of elderly groups (MA and ES; Figure 1b) showed a significant interaction between the activity and sampling time factors [*F*(1.16) = 7.853, *P* = 0.0128, partial η^2^ = 0.3292], with a main effect of activity [*F*(1.16) = 0.3011, *P* = 0.5908, partial η^2^ = 0.0636] and a main effect of sampling time [*F*(1.16) = 26.59, *P* < 0.0001, partial η^2^ = 0.6243]. The *post hoc* test multiple comparisons showed a significant difference between AM and PM samples, with higher AM levels than PM levels in the MA group (Mean diff. = 0.1478, 95% CI = 0.08288 to 0.2128, *P* < 0.0001) and no significant difference between AM and PM in the ES group (Mean diff. = 0.04373, 95% CI = −0.02124 to 0.1087, *P* = 0.2309).

**FIGURE 1:**
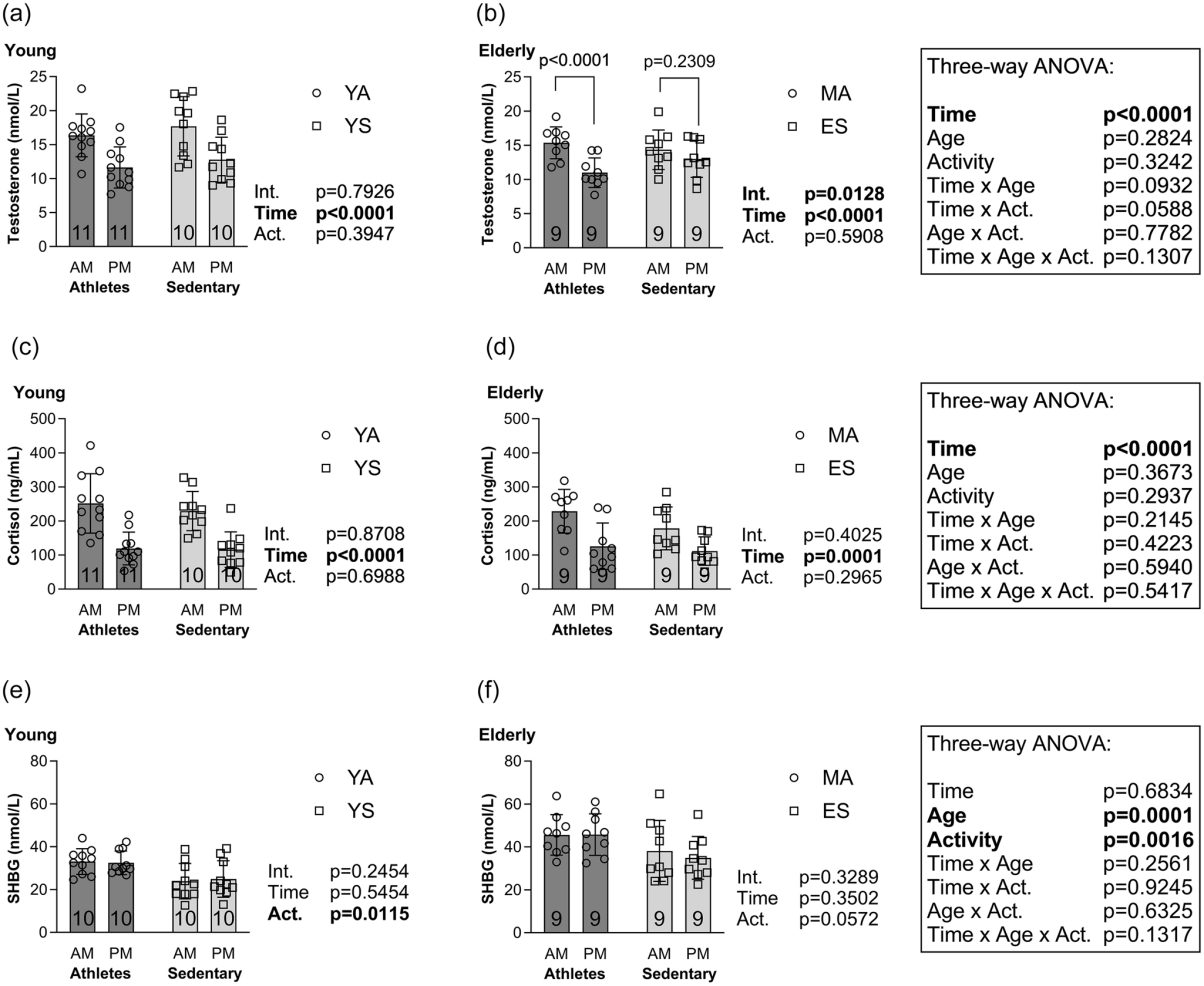
Measured parameters of blood sample analysis. (a) Testosterone (nmol/L) of young participants (YA *n* = 11, YS *n* = 10, MA *n* = 9 and ES *n* = 9; main effect of time, *P* < 0.0001; main effect of activity, *P* = 0.3947; interaction, *P* = 0.7926). (b) Testosterone (nmol/L) of elderly participants (YA *n* = 11, YS *n* = 10, MA *n* = 9 and ES *n* = 9; main effect of time, *P* < 0.0001; main effect of activity, *P* = 0.5908; interaction, *P* = 0.0128; *post hoc* results: MA AM–MA PM, *P* < 0.0001; and ES AM–ES PM, *P* = 0.2309). (c) Cortisol (ng/mL) of young participants (YA *n* = 11, YS *n* = 10, MA *n* = 9 and ES *n* = 9; main effect of time, *P* < 0.0001; main effect of activity, *P* = 0.6988; interaction, *P* = 0.8708). (d) Cortisol (ng/mL) of elderly participants (YA *n* = 11, YS *n* = 10, MA *n* = 9 and ES *n* = 9; main effect of time, *P* = 0.0001; main effect of activity, *P* = 0.2965; interaction, 0.4025). (e) Sex hormone binding globulin (SHBG) (nmol/L) of young participants (YA *n* = 10, YS *n* = 10, MA *n* = 9 and ES *n* = 9; main effect of time, *P* = 0.5454; main effect of activity, *P* = 0.0115; interaction, *P* = 0.2454). (f) SHBG (nmol/L) of elderly participants (YA *n* = 10, YS *n* = 10, MA *n* = 9 and ES *n* = 9; main effect of time, *P* = 0.3502; main effect of activity, *P* = 0.0572; interaction, *P* = 0.3289). Values are expressed as the mean ± SD. The *n* values represent the number of participants in each group and are displayed at the base of corresponding column. Results of two‐way RM ANOVA are listed in the tables beside corresponding graphs. The lines express the results of Bonferroni's multiple comparisons test. Results of a three‐way mixed design ANOVA are listed in the tables on the right side of the figure. YA‐ young athletes, YS‐ young sedentary, MA‐ master athletes, ES‐ elderly sedentary, Int.‐ interaction, Act.‐ Activity, AM‐ morning samples, PM‐ afternoon samples

The three‐way ANOVA of cortisol levels showed a significant main effect of sampling time factor (Table 1), with concentrations decreasing from AM to PM in each participant group. The results of two‐way RM ANOVA within both young (Figure 1c) and elderly (Figure 1d) groups revealed a significant main effect of the time factor [*F*(1.19) = 84.90, *P* < 0.0001, partial η^2^ = 0.8171 and *F*(1.16) = 25.65, *P* = 0.0001, partial η^2^ = 0.6159, respectively] showing a decrease in PM levels. Non‐significant results were obtained in both age groups in the factor of activity [young: *F*(1.19) = 0.1544, *P* = 0.6988, partial η^2^ = 0.0224; elderly: *F*(1.16) = 1.165, *P* = 0.2965, partial η^2^ = 0.1139] and the interaction [young: *F*(1.19) = 0.02719, *P* = 0.8708, partial η^2^ = 0.0014; elderly: *F*(1.16) = 0.7396, *P* = 0.4025, partial η^2^ = 0.0442].

One of the participants in the YA group was identified as a statistical outlier for SHBG and was removed from the analysis and from the calculations of the derived parameters of free androgen index, free testosterone and biologically available testosterone (YA *n* = 10, YS *n* = 10, MA *n* = 9 and ES *n* = 9). The three‐way ANOVA of SHBG resulted in significant main effects of age and activity (Table 1), both leading to a value increase. The two‐way RM ANOVA within the young groups (Figure 1e) resulted in a significant main effect of activity [*F*(1.18) = 7.926, *P* = 0.0115, partial η^2^ = 0.9145], with YA reaching higher levels than YS and with a non‐significant effect of the sampling time factor [*F*(1.18) = 0.3799, *P* = 0.5454, partial η^2^ = 0.0207] or the interaction between the factors [*F*(1.18) = 1.442, *P* = 0.2454, partial η^2^ = 0.0742]. The two‐way RM ANOVA of the elderly groups (Figure 1f) showed no significant main effects of the sampling time factor [*F*(1.16) = 0.9247, *P* = 0.3506, partial η^2^ = 0.0546], activity factor [*F* (1.16) = 4.201, *P* = 0.0572, partial η^2^ = 0.7893] and their interaction [*F*(1.16) = 1.013, *P* = 0.3291, partial η^2^ = 0.0596].

For biologically available testosterone, the three‐way ANOVA resulted in a significant main effect of all three factors (Table 1), all leading to a decrease in level. For the free androgen index and free testosterone, there was also a significant interaction between the three factors of sampling time, age and activity (Table 1), showing that ES did not manifest the significant concentration decrease from AM to PM that was present in the MA, YA and YS groups. The results of *post hoc* test comparisons are listed in Table 2.

The two‐way RM ANOVA within the young groups for the free androgen index (Figure 2a) resulted in significant main effects of time [*F*(1.18) = 34.60, *P* < 0.0001, partial η^2^ = 0.6578] and activity [*F*(1.18) = 10.95, *P* = 0.0039, partial η^2^ = 0.7273], with both factors decreasing the values. The interaction between factors was non‐significant [*F*(1.18) = 0.1970, *P* = 0.6625, partial η^2^ = 0.0108]. The two‐way RM ANOVA within the elderly groups (Figure 2b) resulted in a significant interaction [*F*(1.16) = 8.480, *P* = 0.0102, partial η^2^ = 0.3464] and both main effects [time: *F*(1.16) = 13.08, *P* = 0.0023, partial η^2^ = 0.4498; and activity: *F*(1.16) = 7.080, *P* = 0.0171, partial η^2^ = 0.6655]. The *post hoc* comparisons showed significantly lower levels in PM compared with AM in the MA group (Mean diff. = 0.1485, 95% CI = 0.06894 to 0.2280, *P* = 0.0006) and no difference in the ES group (Mean diff. = 0.01602, 95% CI = −0.06352 to 0.09556, *P *> 0.9999).

**FIGURE 2:**
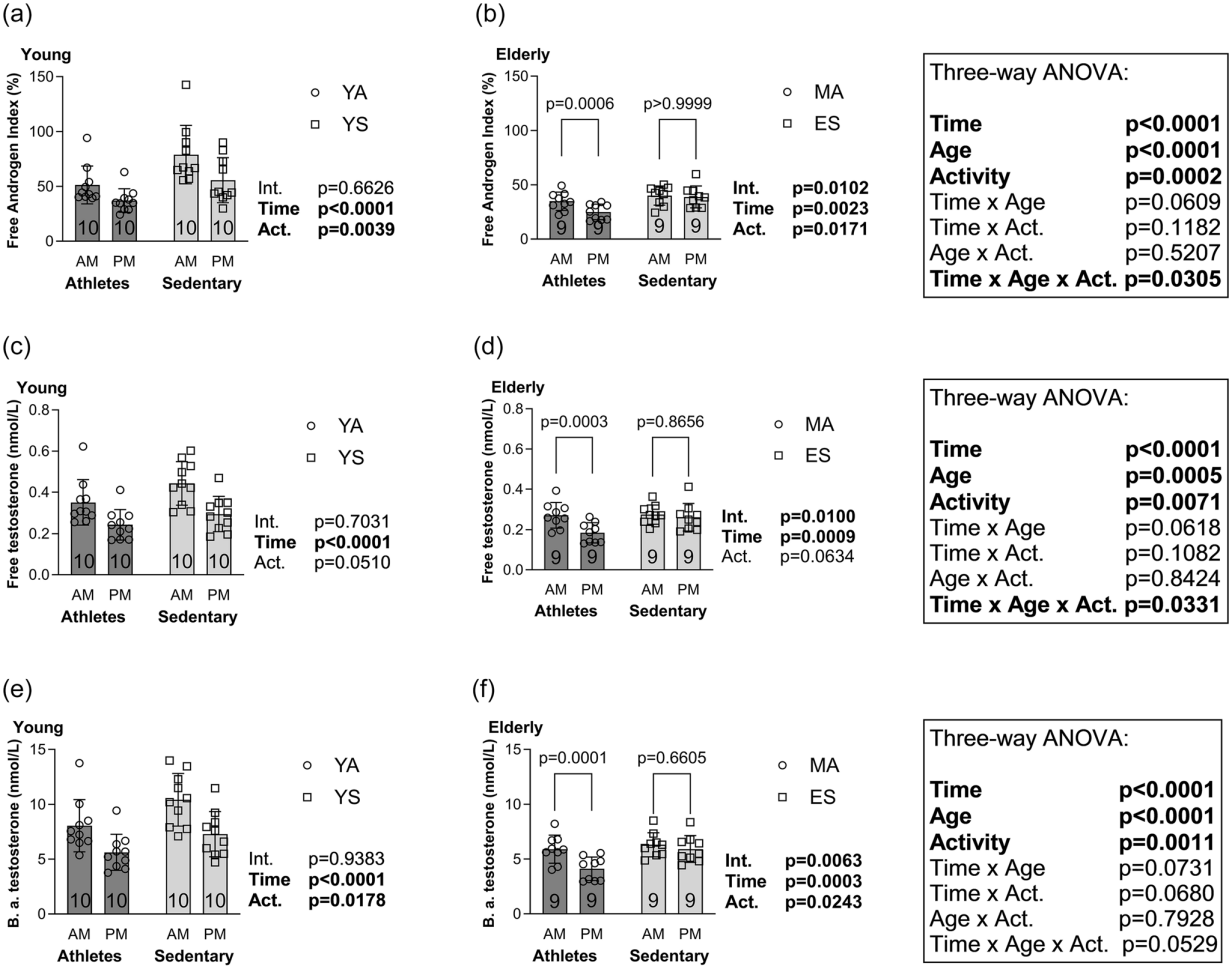
Calculated parameters of blood sample analysis. (a) Free androgen index (as a percentage) of young participants (YA *n* = 10, YS *n* = 10, MA *n* = 9 and ES *n* = 9; main effect of time, *P* < 0.0001; main effect of activity, *P* = 0.0039; interaction, *P* = 0.6626). (b) Free androgen index (as a percentage) of elderly participants (YA *n* = 10, YS *n* = 10, MA *n* = 9 and ES *n* = 9; main effect of time, *P* = 0.0023; main effect of activity, *P* = 0.0171; interaction, *P* = 0.0102; *post hoc* results: MA AM–MA PM, *P* = 0.0006; ES AM–ES PM, *P *> 0.9999). (c) Free testosterone (nmol/L) of young participants (YA *n* = 10, YS *n* = 10, MA *n* = 9 and ES *n* = 9; main effect of time, *P* < 0.0001; main effect of activity, *P* = 0.0510; interaction, *P* = 0.7031). (d) Free testosterone (nmol/L) of elderly participants (YA *n* = 10, YS *n* = 10, MA *n* = 9 and ES *n* = 9; main effect of time, *P* = 0.0009; main effect of activity, *P* = 0.0634; interaction, *P* = 0.0100; *post hoc* results: MA AM–MA PM, *P* = 0.0003; ES AM–ES PM, *P* = 0.8656). (e) Biologically available testosterone (nmol/L) of young participants (YA *n* = 10, YS *n* = 10, MA *n* = 9 and ES *n* = 9; main effect of time, *P* < 0.0001; main effect of activity, *P* = 0.0178; interaction, *P* = 0.9383). (f) Biologically available testosterone (nmol/L) of elderly participants (YA *n* = 10, YS *n* = 10, MA *n* = 9 and ES *n* = 9; main effect of time, *P* = 0.0003; main effect of activity, *P* = 0.0243; interaction, *P* = 0.0063; *post hoc* results: MA AM–MA PM, *P* = 0.0001; ES AM–ES PM, *P* = 0.6605). Values are expressed as the mean ± SD. The *n* values represent the number of participants in each group and are displayed at the base of corresponding column. Results of two‐way RM ANOVA are listed in the tables beside corresponding graphs. The lines express the results of Bonferroni's multiple comparisons test. Results of a three‐way mixed design ANOVA are listed in the tables on the right side of the figure. YA‐ young athletes, YS‐ young sedentary, MA‐ master athletes, ES‐ elderly sedentary, AM‐ morning samples, PM‐ afternoon samples, Int.‐ interaction, Act.‐ Activity, B.a.‐ biologically available

For free testosterone, the two‐way RM ANOVA within the young groups (Figure 2c) resulted in a significant main effect of time [*F*(1.18) = 39.28, *P* < 0.0001, partial η^2^ = 0.6857], with decreased PM values. The main effect of activity [*F*(1.18) = 4.371, *P* = 0.0510, partial η^2^ = 0.3988] and the interaction between the factors [*F*(1.18) = 0.1499, *P* = 0.7031, partial η^2^ = 0.0083] remained non‐significant. The two‐way RM ANOVA within the elderly groups (Figure 2d) resulted in a significant interaction between the factors [*F*(1.16) = 8.532, *P* = 0.0100, partial η^2^ = 0.3478] and the main effect of time [*F*(1.16) = 16.48, *P* = 0.0009, partial η^2^ = 0.5073]. The main effect of activity [*F*(1.16) = 3.979, *P* = 0.0634, partial η^2^ = 0.4112] remained non‐significant. The *post hoc* comparisons showed significantly lowered levels in PM compared with AM in the MA group (Mean diff. = 0.1715, 95% CI = 0.08558 to 0.2574, *P* = 0.0003) and no difference in the ES group (Mean diff. = 0.02796, 95% CI = −0.05797 to 0.1139, *P* = 0.8656).

For biologically available testosterone, the two‐way RM ANOVA within the young groups (Figure 2e) showed significant main effects of time [*F*(1.18) = 37.17, *P* < 0.0001, partial η^2^ = 0.6738] and activity [*F*(1.18) = 6.802, *P* = 0.0178, partial η^2^ = 0.5176], with both factors leading to a decrease in the value. The interaction between the factors was non‐significant [*F*(1.18) = 0.006252, *P* = 0.9379, partial η^2^ = 0.0003]. The two‐way RM ANOVA within the elderly groups (Figure 2f) showed a significant interaction between the factors [*F*(1.16) = 9.866, *P* = 0.0063, partial η^2^ = 0.3814] and main effects of both time [*F*(1.16) = 20.80, *P* = 0.0003, partial η^2^ = 0.5652] and activity [*F*(1.16) = 6.182, *P* = 0.0243, partial η^2^ = 0.5787]. The *post hoc* comparisons showed significantly lowered levels in PM compared with AM in the MA group (Mean diff. = 0.1609, 95% CI = 0.08787 to 0.2340, *P* = 0.0001) and no difference in the ES group (Mean diff. = 0.02967, 95% CI = −0.04340 to 0.1028, *P* = 0.6605).

Evaluation of the estradiol levels (Figure 3) by a two‐way ANOVA resulted in non‐significant main effects of both the age factor [*F*(1.41) = 0.05190, *P* = 0.8209, partial η^2^ = 0.0013] and the activity factor [*F*(1.41) = 3.801, partial η^2^ = 0.0849] and a non‐significant interaction between the factors [*F*(1.41) = 3.315, *P* = 0.0760, partial η^2^ = 0.0748].

**FIGURE 3:**
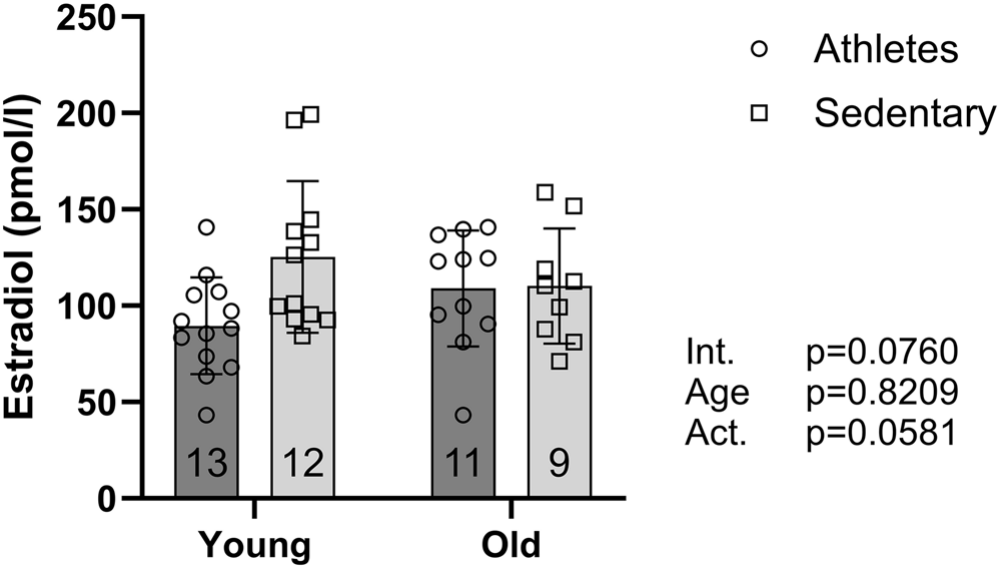
Measured levels of estradiol (pmol/l) in morning blood venous samples. YA *n* = 13, YS *n* = 12, MA *n* = 11 and ES *n* = 9; main effect of age, *P* = 0.8209; main effect of activity, *P* = 0.0581; interaction, *P* = 0.0760). Values are expressed as the mean ± SD. The *n* values represent the number of participants in each group and are displayed at the base of corresponding column. Results of two‐way ANOVA are listed in the table. YA‐ young athletes, YS‐ young sedentary, MA‐master athletes, ES‐elderly sedentary, Int.‐ interaction, Act.‐ activity

#### 3.1.1 Anthropometry

The mean values of age, body mass and height of the participants in each of the study groups are presented in Table 3.

**TABLE 3:** Anthropometric characteristics of study groups of participants.

Parameter	Young athletes (YA)	Young sedentary (YS)	Master athletes (MA)	Elderly sedentary (ES)
Age, years	27.85 ± 2.51	25.75 ± 2.34	69.82 ± 4.00	71.33 ± 4.03
Body mass, kg	74.04 ± 6.49	90.85 ± 16.43	70.77 ± 9.40	81.43 ± 8.38
Height, cm	182.45 ± 4.97	180.87 ± 5.17	171.32 ± 3.56	171.44 ± 5.49
Number of participants, *n*	13	12	11	9

*Note*: Values are expressed as the mean ± SD. The *n* values represent the number of participants in each of the groups.

Regarding BMI (Figure 4a), two‐way ANOVA revealed a significant main effect of activity [*F*(1.41) = 23.48, *P* < 0.0001, partial η^2^ = 0.364], with lower values in the athletic groups. The main effect of age [*F*(1.41) = 0.8081, *P* = 0.3739, partial η^2^ = 0.0193] and the interaction between the factors [*F*(1.41) = 1.025, *P* = 0.3172, partial η^2^ = 0.0244] were non‐significant.

**FIGURE 4:**
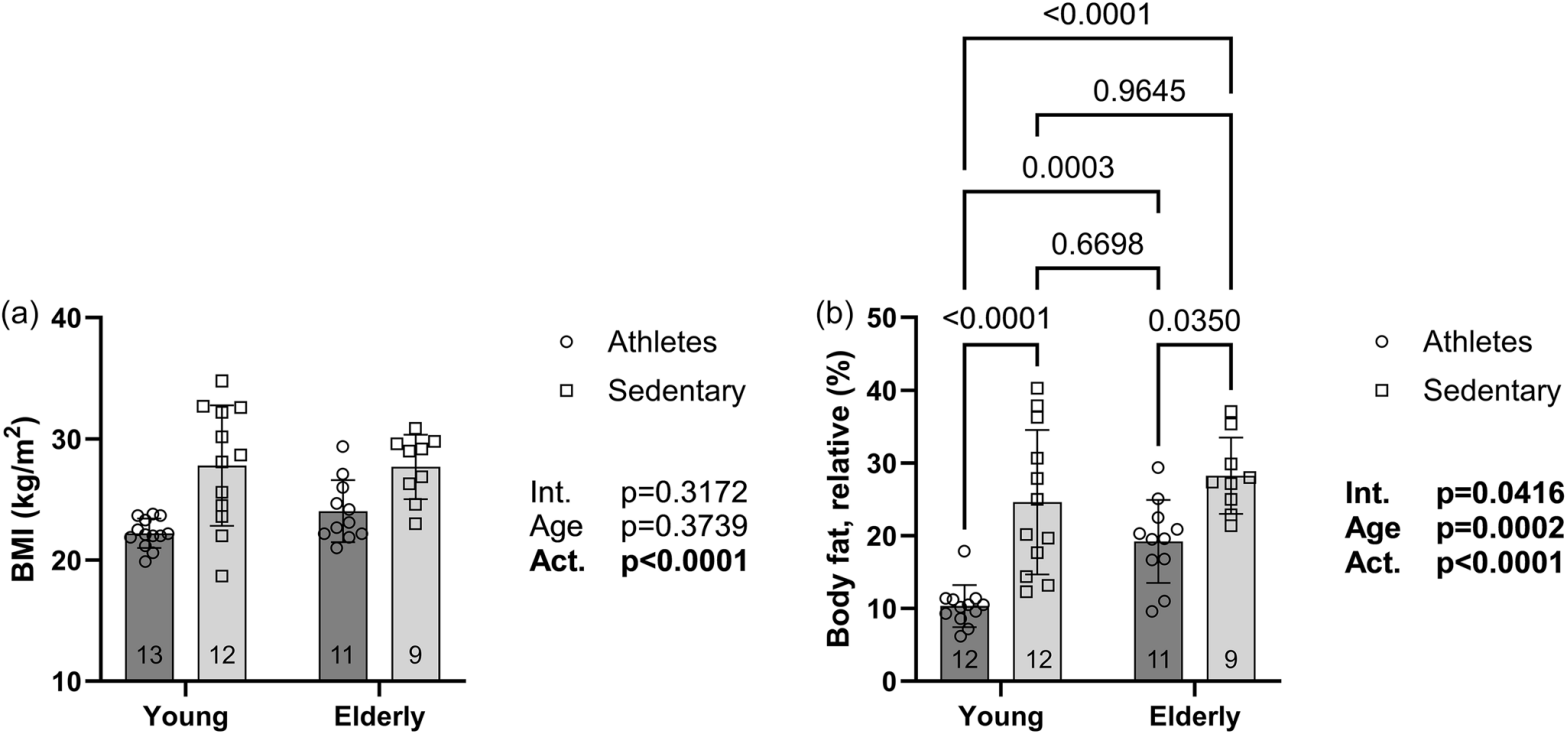
Anthropometric measurements by impedance‐based digital scale. (a) Body mass index value (YA *n* = 13, YS *n* = 12, MA *n* = 11 and ES *n* = 9; main effect of age, *P* = 0.3739; main effect of activity, *P* < 0.0001; interaction, *P* = 0.3172). (b) Body fat, relative (as a percentage) (YA *n* = 12, YS *n* = 12, MA *n* = 11 and ES *n* = 9; main effect of age, *P* = 0.0002; main effect of activity, *P* < 0.0001; interaction, *P* = 0.0416; *post hoc* tests results: YA–YS, *P* < 0.0001; YA–MA, *P* = 0.0003; YA–ES, *P* < 0.0001; YS–MA, *P* = 0.6698; YS–ES, *P* = 0.9654; MA–ES, *P* = 0.0350). Values are expressed as the mean ± SD. The *n* values represent the number of participants in each group and are displayed at the base of corresponding column. Results of two‐way ANOVA are listed in the tables beside corresponding graphs. The lines express the results of Bonferroni's multiple comparisons test. YA‐ young athletes, YS‐ young sedentary, MA‐ master athletes, ES‐ elderly sedentary, Int.‐ interaction, Act.‐ activity

Regarding relative body fat (Figure 4b), one participant from the YA group was identified as a statistical outlier at baseline (*q* = 0.1%; YA *n* = 12). The main effect of activity was significant [*F*(1.40) = 41.18, *P* < 0.0001, partial η^2^ = 0.507], as were the main effect of age [*F*(1.40) = 17.47, *P* = 0.0002, partial η^2^ = 0.304] and their interaction [*F*(1.40) = 4.430, *P* = 0.0416, partial η^2^ = 0.0997]. *Post hoc* comparisons showed significantly lower values in YA compared with YS (Mean diff. = −0.3573, 95% CI = −0.5139 to −0.2008, *P* < 0.0001), YA compared with MA (Mean diff. = −0.2636, 95% CI = −0.4236 to −0.1035, *P* = 0.0003), YA compared with ES (Mean diff. = −0.4444, 95% CI = −0.6135 to −0.2753, *P* < 0.0001) and MA compared with ES (Mean diff. = −0.1808, 95% CI = −0.3532 to 0.0085, *P* = 0.0350) and no significant difference between YS and MA (Mean diff. = 0.0938, 95% CI = −0.06625 to 0.2538, *P* = 0.6698) and between YS and ES (Mean diff. = −0.08704, 95% CI = −0.2561 to 0.08203, *P* = 0.9645).

#### 3.1.2 Muscle strength

According to two‐way ANOVA results, all the muscle strength parameters decreased with increasing age: Ext. Max [Figure 5a; *F*(1.41) = 58.06, *P* < 0.0001, partial η^2^ = 0.586], Flex. Max [Figure 5b; *F*(1.41) = 56.93, *P* < 0.0001, partial η^2^ = 0.581], RTE [Figure 5c; *F*(1.41) = 52.37, *P* < 0.0001, partial η^2^ = 0.561] and RTF [Figure 5d; *F*(1.41) = 42.07, *P* < 0.0001, partial η^2^ = 0.507]. The main effect of activity factor in all the muscle strength parameters was non‐significant [Ext. Max: *F*(1.41) = 1.224, *P* = 0.2712, partial η^2^ = 0.0295; Flex. Max: *F*(1.41) = 2.689, *P* = 0.1087, partial η^2^ = 0.0615; RTE: *F*(1.41) = 0.739, *P* = 0.3951, partial η^2^ = 0.0177; and RTF *F*(1.41) = 0.406, *P* = 0.528, partial η^2^ = 0.00980] and the interactions between the factors were also non‐significant [Ext. Max: *F*(1.41) = 0.5916, *P* = 0.4462, partial η^2^ = 0.0142; Flex Max: *F*(1.41) = 0.1697, *P* = 0.6825, partial η^2^ = 0.00403; RTE *F*(1.41) = 0.0724, *P* = 0.7893, partial η^2^ = 0.00176; and RTF *F*(1.41) = 0.2848, *P* = 0.597, partial η^2^ = 0.00689].

**FIGURE 5:**
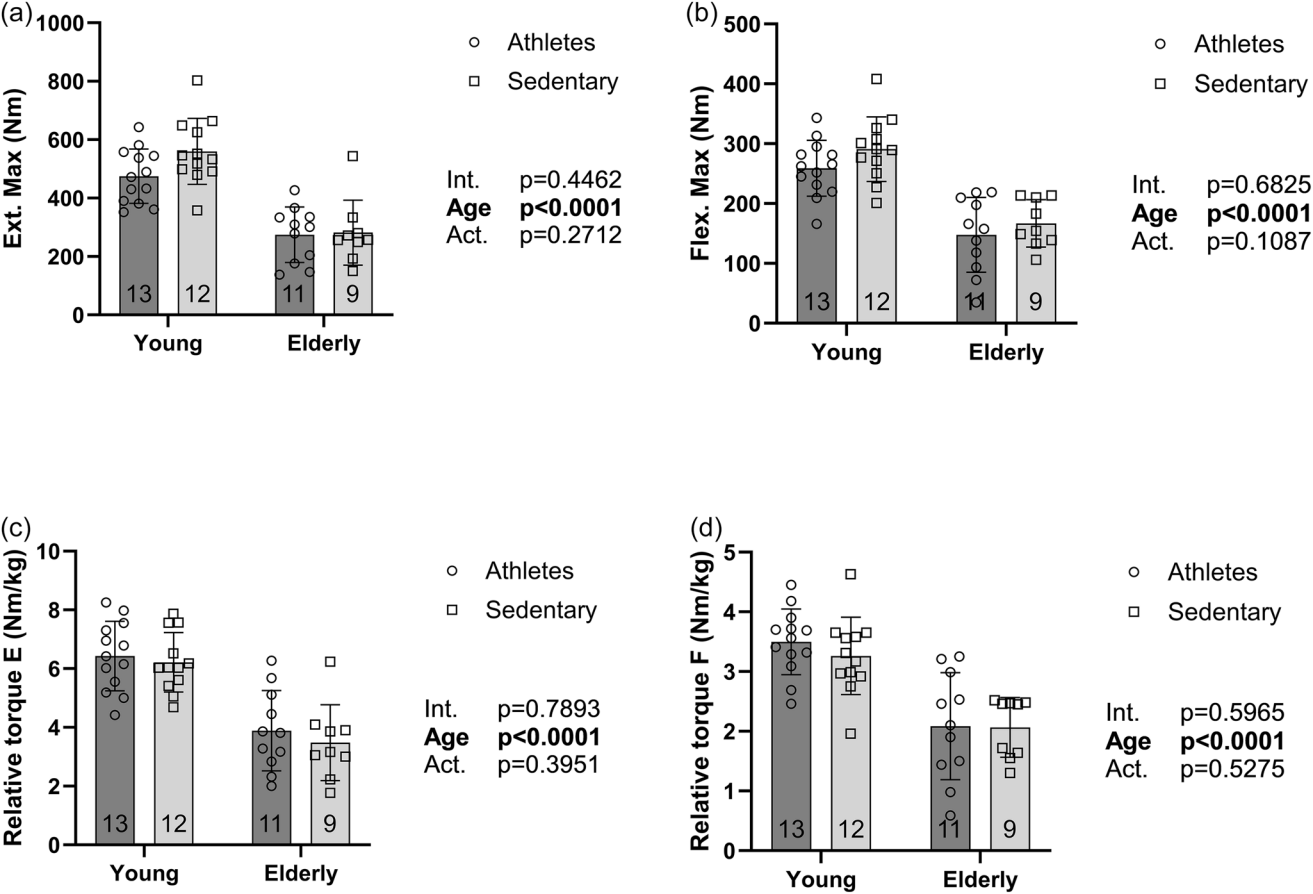
Muscle strength measurements on the knee dynamometer. (a) Maximal extension (Nm) (Ext. Max; YA *n* = 13, YS *n* = 12, MA *n* = 11 and ES *n* = 9; main effect of age, *P* < 0.0001; main effect of activity, *P* = 0.2712; interaction, *P* = 0.4462). (b) Maximal flexion (Nm) (Flex. Max; YA *n* = 13, YS *n* = 12, MA *n* = 11 and ES *n* = 9; main effect of age, *P* < 0.0001; main effect of activity, *P* = 0.1087; interaction, *P* = 0.6825). (c) Relative torque in knee isometric extension (Nm/kg) (RTE; YA *n* = 13, YS *n* = 12, MA *n* = 11 and ES *n* = 9; main effect of age, *P* < 0.0001; main effect of activity, *P* = 0.3951; interaction, *P* = 0.7893). (d) Relative torque in knee isometric flexion (Nm/kg) (RTF; YA *n* = 13, YS *n* = 12, MA *n* = 11 and ES *n* = 9; main effect of age, *P* < 0.0001; main effect of activity, *P* = 0.5275; interaction, *P* = 0.5965). Values are expressed as the mean ± SD. The *n* values represent the number of participants in each group and are displayed at the base of the corresponding column. Results of two‐way ANOVA are listed in the tables beside corresponding graphs. The lines express the results of Bonferroni's multiple comparisons test. YA‐ young athletes, YS‐ young sedentary, MA‐ master athletes, ES‐ elderly sedentary, Int.‐ interaction, Act.‐ activity

#### 3.1.3 Ergometry

Four of the elderly participants were not capable of finishing the ergometry testing at the set intensity and duration protocol, and the testing was stopped by the attending medical professional owing to potentially clinically relevant ECG findings, hence the number of participants in these groups was reduced (MA *n* = 9 and ES *n* = 7).

The HR Max two‐way ANOVA results (Figure 6a) showed a significant interaction between the factors [*F*(1.37) = 11.31, *P* = 0.0018, partial η^2^ = 0.234], with a significant main effect of age [*F*(1.37) = 116.2, *P* < 0.0001, partial η^2^ = 0.759] and a non‐significant main effect of activity [*F*(1.37) = 3.253, *P* = 0.0794, partial η^2^ = 0.0808]. The *post hoc* comparisons showed significantly lower values in the YA compared with the YS group (Mean diff. = −17.04, 95% CI = −28.47 to −5.603, *P* = 0.0011) but no statistically significant difference between the MA and ES groups (Mean diff. = 5.143, 95% CI = −9.252 to 19.54, *P *> 0.9999). Significantly lower values were also observed in MA compared with YA (Mean diff. = 24.46, 95% CI = 12.08 to 36.85, *P* < 0.0001), ES compared with YA (Mean diff. = 29.60, 95% CI = 16.21 to 43.00, *P* < 0.0001), MA compared with YS (Mean diff. = 41.50, 95% CI = 28.90 to 54.10, *P* < 0.0001) and ES compared with YS (Mean diff. = 46.64, 95% CI = 35.26 to 58.03, *P* < 0.0001).

**FIGURE 6:**
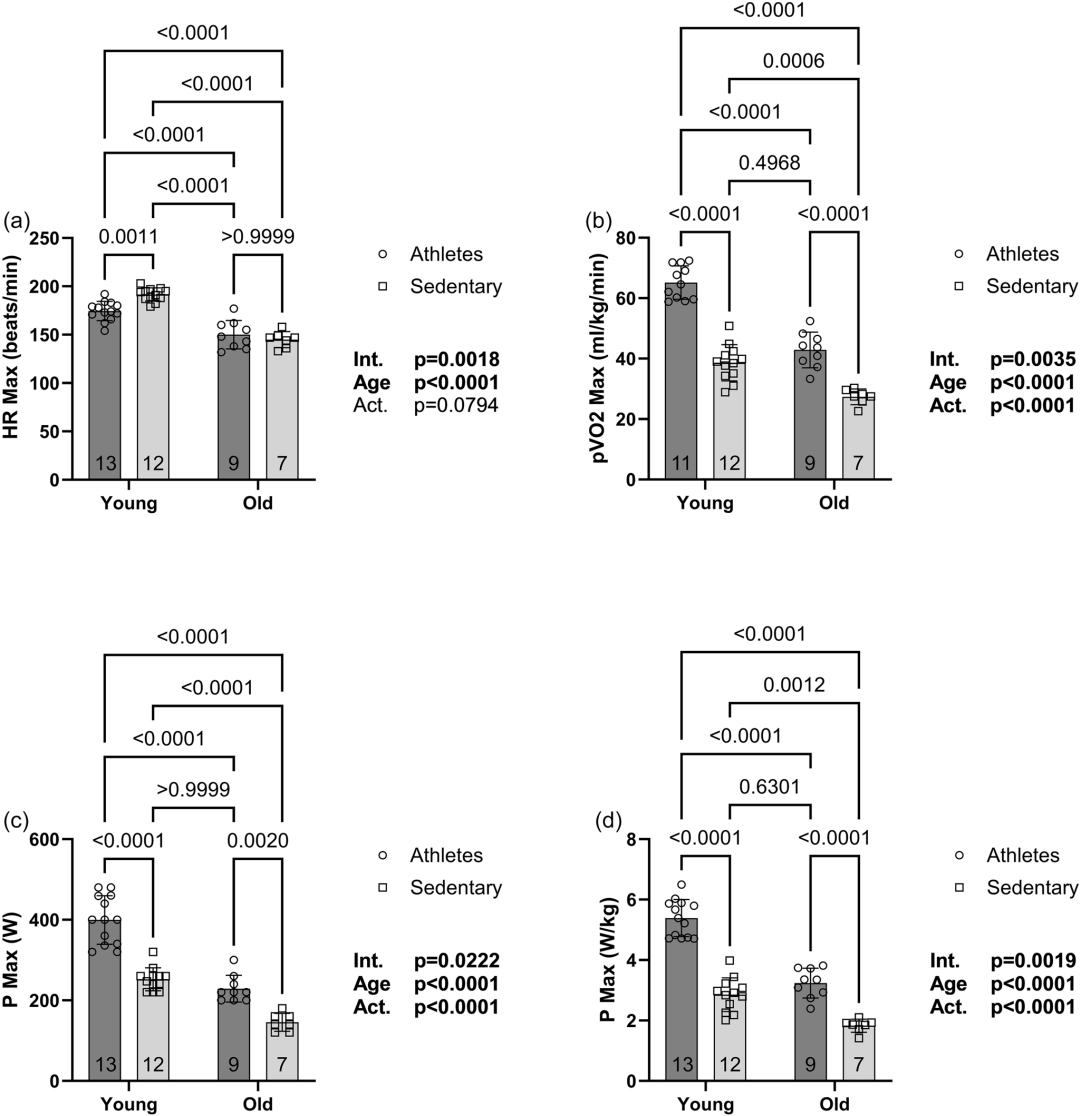
Parameters of cycle ergometry testing. (a) Maximal heart rate (beats/min) (HR Max; YA *n* = 13, YS *n* = 12, MA *n* = 9 and ES *n* = 7; main effect of age, *P* < 0.0001; main effect of activity, *P* = 0.0794; interaction, *P* = 0.0018; *post hoc* test results: YA–YS, *P* = 0.0011; YA–MA, *P* < 0.0001; YA–ES, *P* < 0.0001; YS–ES, *P* < 0.0001; MA–ES, *P *> 0.9999; YS–MA, *P* < 0.0001). (b) Predicted maximal oxygen consumption relative to body mass (ml/kg/min) ($p{\dot{V}}_{O_{2}\max}$; YA *n* = 11, YS *n* = 12, MA *n* = 9 and ES *n* = 7; main effect of age, *P* < 0.0001; main effect of activity, *P* < 0.0001; interaction, *P* = 0.0035; *post hoc* test results: YA–YS, *P* < 0.0001; YA–MA, *P* < 0.0001; YA–ES, *P* < 0.0001; YS–ES, *P* = 0.0006; MA–ES, *P* < 0.0001; YS–MA, *P* = 0.497). (c) Peak power output (W) (P Max; YA *n* = 13, YS *n* = 12, MA *n* = 9 and ES *n* = 7; main effect of age, *P* < 0.0001; main effect of activity, *P* < 0.0001; interaction, 0.0222; *post hoc* test results: YA–YS, *P* < 0.0001; YA–MA, *P* < 0.0001; YA–ES, *P* < 0.0001; YS–ES, *P* < 0.0001; MA–ES, *P* = 0.0020; YS–MA, *P *> 0.9999). (d) Peak power output relative to body mass (W/kg) (P Max relative to body mass; YA *n* = 13, YS *n* = 12, MA *n* = 9 and ES *n* = 7; main effect of age, *P* < 0.0001; main effect of activity, *P* < 0.0001; interaction, *P* = 0.0019; *post hoc* test results: YA–YS, *P* < 0.0001; YA–MA, *P* < 0.0001; YA–ES, *P* < 0.0001; YS–ES, *P* = 0.0012; MA–ES, *P* < 0.0001; YS–MA, *P* = 0.6301). Values are expressed as the mean ± SD. The *n* values represent the number of participants in each group and are displayed at the base of the corresponding column. Results of two‐way ANOVA are listed in the tables beside corresponding graphs. The lines express the results of Bonferroni's multiple comparisons test. YA‐ young athletes, YS‐ young sedentary, MA‐ master athletes, ES‐ elderly sedentary, Int.‐ interaction, Act.‐ activity

Regarding $p{\dot{V}}_{O_{2}\max}$ (Figure 6b), two participants in the YA group had to be excluded from the analysis, owing to measurement error (YA *n* = 11, YS *n* = 12, MA *n* = 9 and ES *n* = 7). The main effect of age was significant [*F*(1.35) = 90.61, *P* < 0.0001, partial η^2^ = 0.721], as were the main effect of activity [*F*(1.35) = 142.2, *P* < 0.0001, partial η^2^ = 0.802] and the interaction between factors [*F*(1.35) = 9.817, *P* = 0.0035, partial η^2^ = 0.219]. The *post hoc* multiple comparisons showed significantly higher levels in YA compared with YS (Mean diff. = 25.56, 95% CI = 20.27 to 32.85, *P* < 0.0001), YA compared with MA (Mean diff. = 22.31, 95% CI = 15.54 to 29.09, *P* < 0.0001), YA compared with ES (Mean diff. = 37.82, 95% CI = 30.53 to 45.11, *P* < 0.0001), YS compared with ES (Mean diff. = 11.26, 95% CI = 4.093 to 18.43, *P* = 0.0006) and MA compared with ES (Mean diff. = 15.51, 95% CI = 7.910 to 23.10, *P* < 0.0001), but no statistically significant difference between YS compared with MA (Mean diff. = −4.244, 95% CI = −10.89 to 2.403, *P* = 0.497.

Regarding P max (Figure 6c), there was a significant main effect of age [*F*(1.37) = 106.6, *P* < 0.0001, partial η^2^ = 0.742] and a significant main effect of activity [*F*(1.37) = 73.60, *P* < 0.0001, partial η^2^ = 0.665]. The interaction between factors was significant [*F*(1.37) = 5.695, *P* = 0.0222, partial η^2^ = 0.133]. The *post hoc* multiple comparisons showed significantly higher levels in YA compared with YS (Mean diff. = 147.3, 95% CI = 100.7 to 193.9, *P* < 0.0001), YA compared with MA (Mean diff. = 170.7, 95% CI = 120.3 to 221.2, *P* < 0.0001), YA compared with ES (Mean diff. = 253.9, 95% CI = 199.4 to 308.4, *P* < 0.0001), YS compared with ES (Mean diff. = 106.6, 95% CI = 51.28 to 162.0, *P* < 0.0001) and MA compared with ES (Mean diff. = 83.17, 95% CI = 24.54 to 141.8, *P* = 0.0020), but no statistically significant difference between YS compared with MA (Mean diff. = 23.44, 95% CI = −27.86 to 74.75, *P *> 0.9999).

Regarding P max relative to body mass (Figure 6d), there was a significant main effect of age [*F*(1.37) = 89.12, *P* < 0.0001, partial η^2^ = 0.707] and a significant main effect of activity [*F*(1.37) = 137.3, *P* < 0.0001, partial η^2^ = 0.788]. The interaction between factors was significant [*F*(1.37) = 11.18, *P* = 0.0019, partial η^2^ = 0.232]. The *post hoc* multiple comparisons showed significantly higher levels in YA compared with YS (Mean diff. = 2.537, 95% CI = 1.953 to 3.122, *P* < 0.0001), YA compared with MA (Mean diff. = 2.154, 95% CI = 1.521 to 2.787, *P* < 0.0001), YA compared with ES (Mean diff. = 3.564, 95% CI = 2.880 to 4.249, *P* < 0.0001), YS compared with ES (Mean diff. = 1.027, 95% CI = 0.3330 to 1.721, *P* = 0.0012) and MA compared with ES (Mean diff. = 1.411, 95% CI = 0.6752 to 2.146, *P* < 0.0001), but no statistically significant difference between the YS compared with MA (Mean diff. = −0.3836, 95% CI = −1.027 to 0.2599, *P* = 0.6301).

## 4 DISCUSSION

The purpose of our study was to evaluate the time‐of‐day‐dependent testosterone secretion in its total, biologically available and free forms in the MA group and its comparison with their sedentary counterparts and young control subjects. Our analysis indicates that long‐term endurance training might be associated with the presence of daily variation in free testosterone in elderly men. In our study, the free testosterone and free androgen index in the ES group did not manifest the PM level decrease that was present in the young groups. However, in the case of MA, the free testosterone and free androgen index daily level variation was significantly present, comparable to the younger participants.

The parameters of total testosterone and biologically available testosterone did not result in a significant interaction between the age, activity and time‐of‐the‐day factors based on the three‐way ANOVA, indicating that differences in their daily variation according to activity status were not statistically different between the young and elderly groups. However, after performing additional, biologically targeted follow‐up statistical analysis of two‐way RM ANOVAs to focus on data within the separate age groups, in the elderly population only the MA group showed a significant variation between AM and PM levels, whereas ES levels remained consistent between the two time points.

One of the key implications of these findings is an emphasis on the importance of evaluating the sedentary or active lifestyle in studies of the ageing process. The attenuation of testosterone daily variation is usually described as age dependent, or ageing associated (Basaria & Dobs, 1999; Brambilla et al., 2009), whereas in our study, differences in free testosterone AM–PM variation were observed between elderly men with different physical activity status. Even a recent study analysing diurnal variation of testosterone levels in elderly men did not consider the activity/training status of the participants (Novaes et al., 2024). In general, given that regular physical exercise has a significant impact on physiological systems during ageing, it was suggested by Lazarus et al. (2019) that studies investigating healthy processes of human ageing should consider the detailed physical activity status of the studied individuals. Ignoring the physical activity of participants can increase the risk of interpreting declining physiological parameters as attributable to ageing, whereas they might be caused by physical inactivity or other lifestyle factors (Lazarus et al., 2019).

The SHBG levels were affected by both the age and activity factors, both with a significant increasing effect, accompanied by the decrease in related parameters: free androgen index, free testosterone and biologically available testosterone. Although our results did not show lower total testosterone levels in the young or elderly athletic groups compared with their sedentary counterparts, the athletic participants exhibited lower levels of testosterone in its free or bioavailable form compared with the untrained. In the literature, a rise in SHBG levels during ageing has been reported (Basaria & Dobs, 1999). Exercise training has been reported to have an elevating effect on SHBG levels in elderly sedentary men (Hayes et al., 2015), and elevation of SHBG levels has also been reported associated with male professional endurance runners (Nogal et al., 2023). With regard to the effect of training activity, the elevation of SHBG levels and consequent decrease in free androgen index, free testosterone and biologically available testosterone suggest a potential adverse effect of endurance running. Lower testosterone levels are not uncommon for endurance athletes, and endurance training is often associated with a negative impact on reproductive health, including lowered total and free testosterone levels, as a part of REDS syndrome (Cupka & Sedliak, 2023) or the exercise–hypogonadal condition (Hackney, 2008).

The described relationships of SHBG levels with BMI and fat tissue agree with our results. The BMI differed significantly between the athletic and sedentary groups. According to the WHO classification (‘Obesity: Preventing & Managing the Global Epidemic. Report of a WHO Consultation’, 2000), both athletic groups had an average BMI value of ‘normal weight’ (between 18.5 and 25 kg/m^2^), whereas the non‐athletic groups had average BMI values at the level of ‘pre‐obesity’ or overweight (≥25 kg/m^2^). The athletes also reached significantly lower values in the relative body fat parameter in both age groups. The significant interaction between age and training factors in the relative body fat parameter indicated that the difference between athletic and sedentary participants was smaller in the elderly groups. Our data are in line with meta‐analysis results, which concluded that endurance training might attenuate the age‐related increase in body fat, because master endurance athletes tended to reach lower relative values than those of elderly control individuals but higher than those of young endurance‐trained athletes. Moreover, the MA tend to maintain a similar body fat percentage to untrained young control subjects, with no significant differences between the groups (Mckendry et al., 2018). Based on our results, in which the untrained elderly control group also did not differ statistically from their young counterparts, we conclude that endurance training in MA did not result in maintenance of young‐level body fat values, but rather in an improvement in their own age category.

The cortisol level results showed no effect of age or activity factors within their total values. Daily variation of cortisol levels was present in all the groups of participants, but it was not affected by the age or activity factors. Cortisol circadian rhythms during ageing are often associated with a reduction of the amplitude and a phase shift of acrophase of the rhythm (Hood & Amir, 2017; van Coevorden et al., 1991). It is possible that the results were affected by the broader study protocol, in which the muscle biopsy was scheduled immediately after the morning blood sampling. The expectation of muscle biopsy might have caused a greater stress response in participants, resulting in elevated cortisol levels, as reported before (Sedliak et al., 2007). To assess the effect of training activity on the daily variation of cortisol in ageing men, it is necessary to repeat the study with a different testing protocol, preferably with a larger number of participants.

Estradiol plasma concentration did not differ significantly between the groups of participants. In men, circulating estradiol levels tend to decline with age, based on their dependence on testosterone (Orwoll et al., 2006; Wu et al., 2018), but at the same time they are more stable because of increasing aromatase activity with age and the age‐associated increase in fat mass (Vermeulen et al., 2002).

Muscle strength decreased significantly with advancing age but was not affected by the endurance running training factor. Even with the leg muscle strength measured, both athletes and untrained men reached comparable results. Interestingly, our results are not in line with those of Tøien et al. (2023), who reported higher absolute and normalized strength in endurance‐trained individuals compared with control subjects in both young and elderly groups, measured by leg‐press test, or with results of Aagaard et al. (2007), who described higher values of maximum isometric quadriceps moment in elderly endurance‐trained MA compared with a control group of untrained elderly individuals, measured on an isokinetic dynamometer.

Within the HR Max parameter, we found a decreasing impact of the age factor and a significant interaction between the factors of age and activity. The age‐dependent decrease in HR Max regardless of training activity has already been well described (Valenzuela et al., 2020). In our study, the comparison of young groups showed that the YS reached higher values than the YA group. It is possible either that the testing protocol did not allow the YA participants to reach the HR Max owing to the test duration or that they did not have sufficient strength for this particular protocol.

The values of P Max and P Max relative to body mass decreased significantly with age and were elevated by the training activity. The interaction between these two factors was also significant. The ES group differed significantly from both the YA and YS groups, whereas the MA group did not differ from the YS group, showing values comparable to those observed in sedentary men 20–30 years of age.

Similar results were obtained for $p{\dot{V}}_{O_{2}\max}$, in which MA reached levels closest to those of young, untrained males. However, these results should be interpreted with caution and only for the purpose of the group characterization owing to the selected standardized protocol, estimating the values indirectly from cycling performance in the group of endurance runners. A similar conclusion, that master endurance athletes have comparable $p{\dot{V}}_{O_{2}\max}$ results to younger control subjects and higher than those of elderly control subjects, has been published in a meta‐analysis (Mckendry et al., 2018).

The main limitation of the present study is the low number of participants involved, mainly attributable to the difficulty in recruitment of elderly participants who were able to meet the inclusion and exclusion criteria. Although reporting the levels and daily variation of various hormones, we did not assess every factor that could influence the regulation of these hormones, such as sleep patterns and the chronotype of the participants, recent training load, evaluation of psychological stress, dietary composition or casual alcohol intake. Another possible limitation concerns the use of cycling ergometry to assess cardiovascular fitness. Although this method was chosen by design to enhance feasibility and safety, especially for older individuals, it might have undervalued the aerobic capacity in endurance runners. These athletes generally reach higher ${\dot{V}}_{O_{2}\max}$ values during treadmill exercise compared with cycling, reflecting the specificity of exercise modalities. Furthermore, the set resistance and the gradual increase of load during the testing probably resulted in premature termination of the test in all groups. In the YA group, it is likely that the increasing strength demands of the test did not allow them to achieve their individual maximum heart rate. Additionally, maximal effort ergometry testing in the elderly groups was associated with practical limitations, because four participants were unable to complete the protocol for medical reasons.

## 5 CONCLUSION

Our results indicate that long‐term endurance training might be associated with the presence of daily variation in plasma free testosterone levels and free androgen index in elderly men, in contrast with their sedentary counterparts. Endurance running training is also associated with increased SHBG levels and decreased plasma free testosterone, biologically available testosterone and free androgen index values, suggesting a potential amplification of age‐related changes by the endurance running activity in androgen hormone regulation. In contrast, long‐term endurance running in elderly men is associated with significantly higher mechanical power performance, both in absolute terms and relative to body mass values, compared with their sedentary counterparts, remaining comparable to those observed in healthy young, untrained men.

## AUTHOR CONTRIBUTIONS

Barbora Bartko: data curation, formal analysis, visualization, writing—original draft, writing—review & editing; Ľudmila Oreská: conceptualization, data curation, funding acquisition, investigation, methodology, project administration, writing—review & editing; Gabriel Buzgó: conceptualization, data curation, investigation, methodology, writing—review & editing; Genc Berisha: data curation, formal analysis, investigation, methodology, project administration, validation, writing—original draft; Valentína Zemaníková: data curation, formal analysis, investigation, methodology, project administration, writing—review & editing; Monika Okuliarová: conceptualization, data curation, funding acquisition, investigation, methodology, project administration, resources, supervision, writing—review & editing; Michal Zeman: conceptualization, data curation, funding acquisition, investigation, project administration, resources, supervision, writing—review & editing; Milan Sedliak: conceptualization, data curation, funding acquisition, investigation, project administration, resources, supervision, writing—review & editing. All authors approved the final version of the manuscript and agree to be accountable for all aspects of the work in ensuring that questions related to the accuracy or integrity of any part of the work are appropriately investigated and resolved. All persons designated as authors qualify for authorship, and all those who qualify for authorship are listed.

## CONFLICT OF INTEREST

The authors declare no conflicts of interest.

## GENERATIVE AI STATEMENT

The authors confirm that no artificial intelligence tools, including large language models (LLMs), were used in the drafting or revision of this manuscript. All content was conceived, written and approved solely by the authors.

## Data Availability

The data that support the findings of this study are available from the corresponding author upon reasonable request.
